# Binet’s Tests for Backward Children

**Published:** 1913-03

**Authors:** 


Binet's Tests for Backward Children. Siegfried Block of Brooklyn, *Am. Med.*, Nov., 1912, criticizes them and suggests some modifications. The article cannot well be abstracted, but should be read by those especially interested. The popular notion, due to rather sensational magazine articles, that persons can be accurately graded as 5, 10, 15 years old, mentally, should be corrected. Inattention, accidental ignorance on certain points or equally accidental or useless information on certain others, misunderstanding, especially by those unfamiliar with the language used, may give very misleading ratings. We have seen it stated that an adolescent or an adult should be able to remember and reproduce easily, a series of five arbitrary words. Everyday experience with Federal telephonic numbers of five figures shows that this is not the case.

